# Flexible Quantum-Dot Light-Emitting Diodes Using Embedded Silver Mesh Transparent Electrodes Manufactured by an Ultraprecise Deposition Method

**DOI:** 10.1021/acsomega.3c04601

**Published:** 2023-10-12

**Authors:** Łukasz Witczak, Maciej Chrzanowski, Piotr Sitarek, Mateusz Łysień, Artur Podhorodecki

**Affiliations:** † XTPL SA, Stabłowicka 147, 54-066 Wroclaw, Poland; ‡ Department of Experimental Physics, Faculty of Fundamental Problems of Technology, Wroclaw University of Science and Technology, Wybrzeże Wyspiańskiego 27, 50-370 Wroclaw, Poland; § Institute of Low Temperature and Structure Research, Polish Academy of Sciences, Okólna 2, 50-422 Wroclaw, Poland

## Abstract

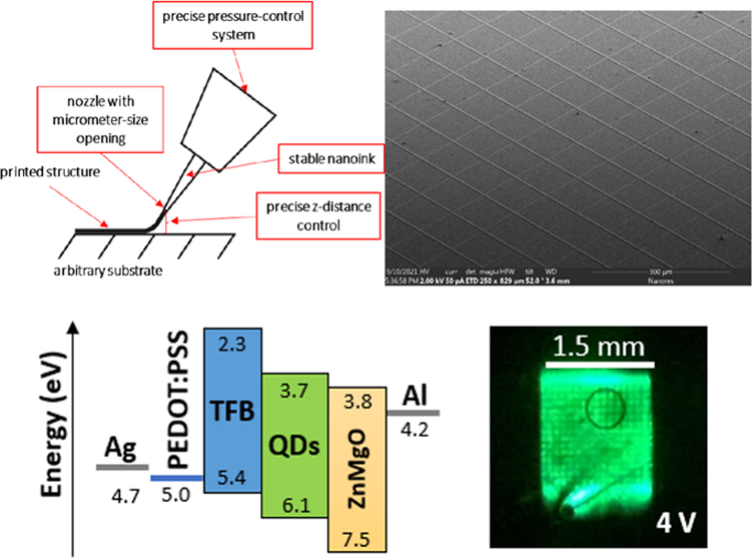

Transparent conductive electrodes (TCEs) fabricated onto flexible substrates are crucial parts of organic-light-emitting diodes (OLEDs), which are vastly utilized for display and lightning applications. Indium tin oxide (ITO), which is so far the most popular material for transparent and conductive electrodes, is found to be an unsuitable candidate for flexible devices mostly due to its brittleness. Here, we present a novel approach for the fabrication of transparent, conductive, and flexible electrodes for optoelectronic applications made of silver metal mesh by an ultraprecise deposition (UPD) method. The fabricated mesh exhibits an 80% (λ = 550 nm) optical transmittance and a sheet resistance of 11 Ω/sq. The Ag-mesh embedded into the polymer is implemented as an anode for a quantum-dot light-emitting diode (QLED) in order to assess its performance. The fabricated QLED is characterized by the maximum external quantum efficiency (EQE) of 2% and a current efficiency (CE) of 6 cd/A, reaching the maximum luminance (*L*) of 3200 cd/m^2^ at a current density of 100 mA/cm^2^. This method shows a fast and relatively simple approach to fabricate optoelectronic devices without the need for special treatment and sophisticated equipment.

## 1 Introduction

The fabrication of transparent conductive electrodes (TCEs) on flexible substrates has garnered increasing attention in recent years due to the demands of the fast-growing optoelectronics industry, especially in the field of flexible (wearable) optoelectronics.^1,2^ TCEs are vastly used as components of flexible solar cells, sensors, and light-emitting diodes (LED), including OLEDs and quantum-dot light-emitting diodes (QLEDs).^2−7^ Indium tin oxide (ITO) is the most popular material for transparent electrodes due to its low sheet resistance and high transmittance. This material is however not a suitable candidate for flexible electrodes mainly due to its brittleness and the scarcity of indium.^8^ For this reason, many studies have been conducted recently to find substitutes for ITO that will have satisfactory flexibility while maintaining reasonable transmittance, conductivity, and production costs. To overcome this issue, the synthesis of cracking-template-based self-formed metal-mesh-based TCEs has been one of the approaches to develop transparent conducting electrodes (TCEs). In this technique, a crackle precursor is deposited (sprayed, poured) onto the substrate. After drying, the precursor leaves cracks on the substrate surface. Subsequently, a metal can be deposited into these cracks by physical vapor deposition, electroless deposition, etc. Then, the templates can be washed away, leaving the metal mesh on the substrate surface. The fabricated TCEs exhibit low sheet resistance (<10 Ω/sq typically) and high transmittance (≈86–90% at 550 nm typically). However, they suffer from several limitations and disadvantages, such as the complexity of template formation, limited material compatibility (between the crackle precursor and metal), material wastage, or inconsistent deposition over large areas.^9^ Besides the cracking-template-based metal mesh, the most popular proposed substitutes for ITO are so far carbon-based materials,^6,7,10^ conducting polymers,^11^ metal grids, metal nanowires,^12−16^ or dielectric/metal/dielectric (DMD) materials.^17−19^

Special attention has been focused on electrodes based on silver and copper grid/mesh as they are considered to be one of the most attractive candidates in terms of their high electrical conductivity, transmittance, and simplicity of preparation process. Hence, they are vastly utilized as mechanically flexible electrodes in OLEDs.^20−22^ The drawbacks include a relatively high contact resistance, high surface roughness, and elevated sintering temperatures (>200 °C), which may be problematic when considering commercial use of such an approach in the OLED industry, for example.

One of the solutions to reduce the roughness of the silver grids and to avoid elevated sintering temperatures of silver acting on the plastic substrate could be transferring the metallic mesh from a solid to a flexible substrate. To achieve this, the silver mesh can be first manufactured onto the glass substrate at which the sintering process takes place and then transferred into the elastic substrate. Subsequently, the flat substrate provides the possibility for embedding a silver grid into the polymer before peeling it off from the glass substrate.^15,16^

**Figure 1:**
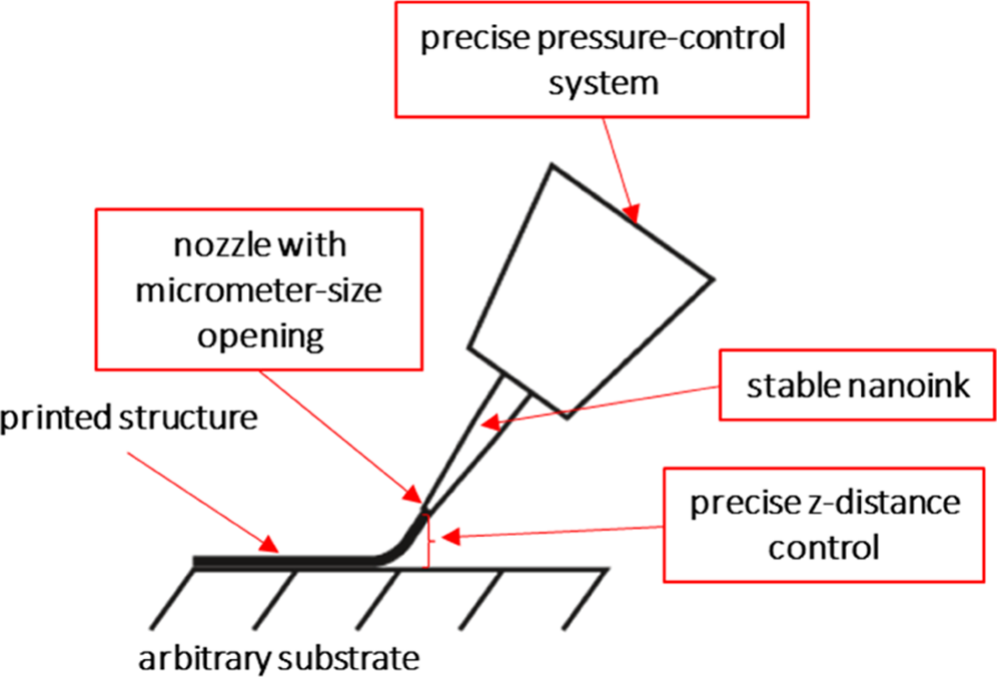
Schematic representation of the UPD technology.

Such a process would be especially interesting if the deposition of the Ag-grid electrode would be performed by a printing technology as this significantly reduces the production costs and simplifies the whole deposition process in contrast to standard vacuum deposition processes, which involve complex fabrication steps. Among various printing methods, the most promising printing technology is a novel ultraprecise deposition (UPD) technology provided by XTPL.^23−25^ UPD allows a direct deposition of highly concentrated silver inks (even 82 wt % metal content) on complex substrates. The printed feature size is as small as 1 μm with an electrical conductivity of up to 45% bulk silver. This method does not require any electric field, which is crucial for the safety of electrical components on the substrate, and is compatible with various types of substrates: conductive or nonconductive, complex morphology. Figure 1 depicts a sketch of the UPD process. The material is directly deposited on the substrate in a continuous manner through a nozzle with a diameter in the range of 0.5–10 μm, which gives the printed feature size in the range of 1–10 μm. The process is governed by pneumatic pressure controlled by a precise pressure-control system. The printing process also requires precise control over the distance between the nozzle and the substrate.

In this paper, we present a novel approach for manufacturing flexible TCEs using a plastic substrate with an embedded silver mesh deposited by the XTPL UPD technique. This Ag metal-mesh TCE was then incorporated into a quantum-dot light-emitting diode (QLED) in order to demonstrate its application and performance.

## 2 Results and Discussion

### 3.1 Ag Metal Mesh

**Figure 2:**
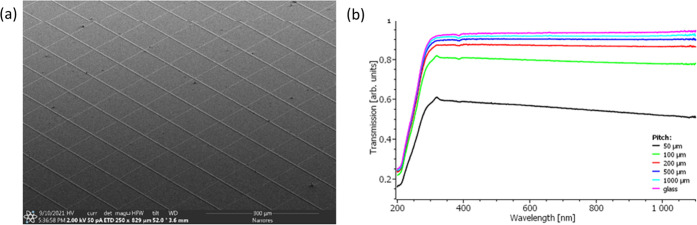
(a) Scanning electron microscopy (SEM) image showing the topology of Ag after thermal sintering on the glass substrate. (b) Optical transmittance of the Ag-mesh on the substrate glass.

Several different silver meshes have been fabricated in terms of their pitch in order to find a mesh with the best trade-off solution between optical transparency and sheet resistance. The width of the lines was fixed at 5 ± 0.5 μm. The single Ag line with a width of approximately 5 μm has an aspect ratio of 1:10. The conductivity of a single Ag line after thermal sintering is 0.04 Ω/μm. The pitch between the lines was constant for each mesh, and the tested pitches are 50, 100, 200, 500, and 1000 μm. Figure 2a depicts the SEM image of the printed Ag-mesh with a pitch of 100 μm. The printed lines preserved their shape and smooth morphology. The measured surface roughness for a single printed Ag line after thermal sintering is 0.03 μm. Figure 2b shows the UV–vis measurements for each printed Ag mesh. The mesh with the smallest pitch has the lowest transmittance along the whole measured spectrum. The reason for this phenomenon is that a smaller pitch results in a denser Ag mesh, and as a result, less light comes through the mesh. From Table 1, it can be concluded as to which line distance (pitch) is the most optimal. With increasing spacing between the printed lines, the optical transmittance increases. However, the sheet resistance decreases. The mesh with the best parameters has a spacing of 100 μm. The sheet resistance is almost four times better than that for a mesh with a pitch of 200 μm, but the optical transmittance decreases by only 7%. This trade-off is the best among the manufactured Ag meshes. Therefore, the Ag mesh with a pitch of 100 μm was used for the fabrication of quantum-dot light-emitting diodes (QLEDs). The thickness of NOA63 varies from 100 to 1000 μm. On NOA63, the sheet resistance of the Ag metal mesh was maintained at the level of 11 Ω/sq. The maximum transmittance is 72%. This means that the transfer of the mesh was successful, and the sheet resistance was not increased.

**Table 1:** Sheet Resistance (Ω/sq) and Optical Transmittance at λ = 550 nm (%) for Printed Silver Meshes in Terms of their Different Pitch (μm) Values^a^

pitch (μm)	sheet resistance (Ω/sq)	optical transmittance at λ = 550 nm (%)
50	1.8	57
100	11	80
200	40	87
500	66	90
1000	165	92

a Optical transmittance of the glass substrate is 93%.

### 3.2 Ag-Mesh QLED

**Figure 3:**
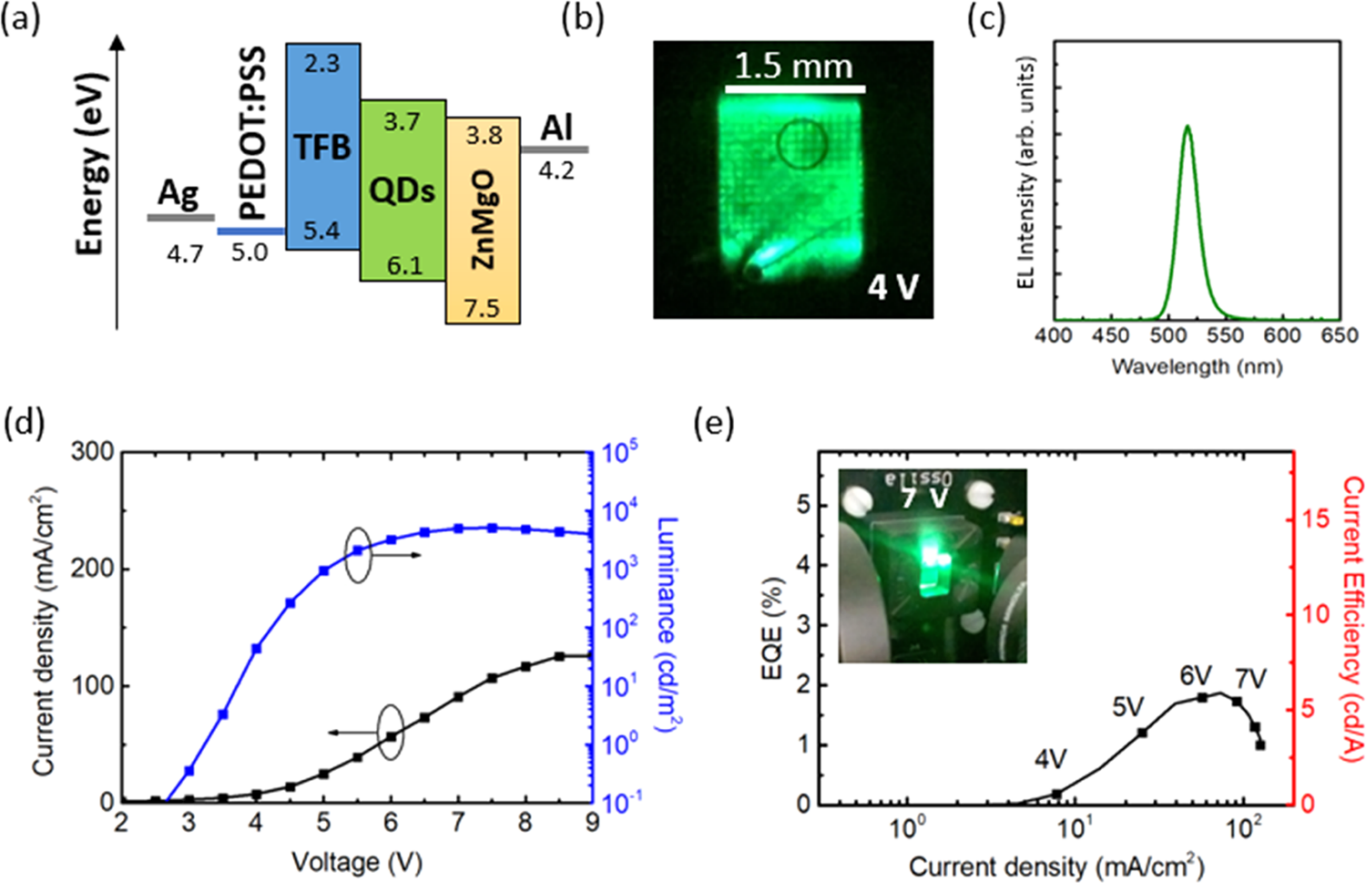
(a) Energetics scheme of the QLED under investigation. (b) Image of the emitting area of the device and the (c) corresponding electroluminescence spectrum. (d) *J*–*V*–*L* and (e) EQE–*J*–CE characteristics of the QLED. The inset shows an image of a pixel area.

**Figure 4:**
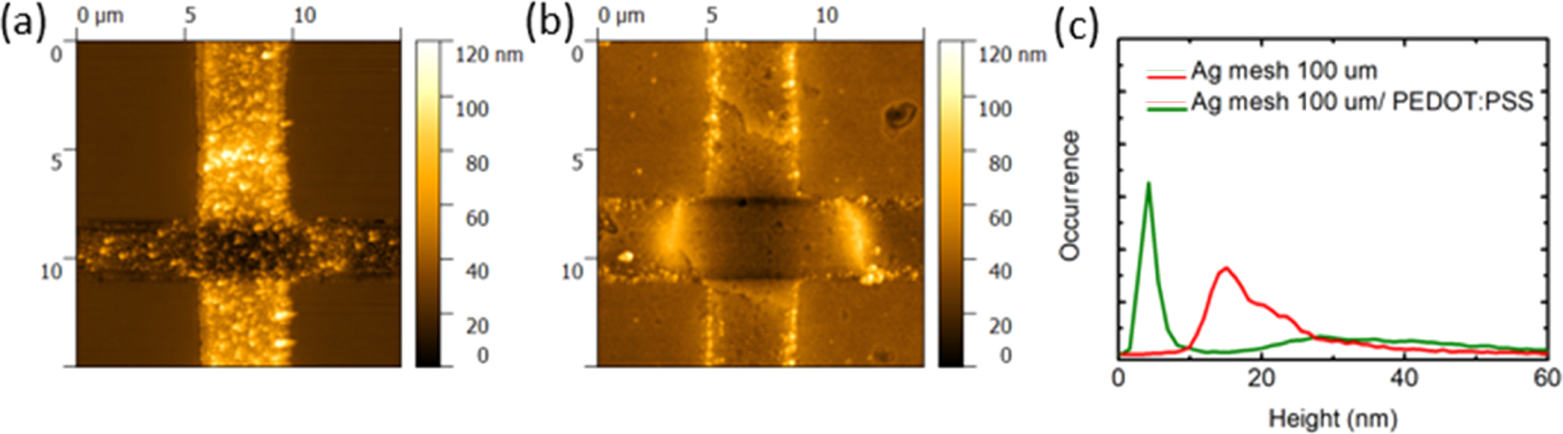
Atomic force microscopy (AFM) images showing the crossing section of the Ag mesh before (a) and after (b) the deposition of the PEDOT:PSS coating. (c) Corresponding height histograms collected at the Ag surface, showing noticeable roughness reduction.

In the next step, the TCE was used to fabricate a quantum-dot light-emitting diode (QLED) with the structure shown in Figure 3a. The device was composed of a flexible-film SU66/Ag metal-mesh TCE integrated with QLED thin films: PEDOT:PSS(50 nm)/TFB(30 nm)/QDs(25 nm)/ZnMgO(45 nm)/Al(100 nm), where the TFB is poly(9,9-dioctylfluorene-*alt*-*N*-(4-*s*-butylphenyl)-diphenylamine). Because of the low resistivity of the used hole injection layer (HIL) polymer (1–10 Ω·cm) compared to that of the standard Al 4083 PEDOT:PSS formulation (500–5000 Ω·cm), we achieved uniform emission with a maximum at 515 nm between metal grid lines, as shown in Figure 3b,3c. The device was characterized by the maximum external quantum efficiency (EQE) of 2% and a current efficiency (CE) of 6 cd/A, reaching the maximum luminance (*L*) of 3200 cd/m^2^ at a current density of 100 mA/cm^2^ (Figure 3d,3e). Importantly, the HIL was deposited without complex transfer techniques but was spin-coated on SU66/Ag-mesh foil. This was possible thanks to the excellent wetting properties of the isopropanol formulation of the used PEDOT:PSS solution without using any further additives. To confirm the sample homogeneity, AFM images of TCE were obtained before and after the deposition of a 50 nm PEDOT:PSS film (Figure 4a,4b). The root-mean-square (RMS) roughness at the surface of the Ag film was reduced from 14 to 5 nm, as indicated by the obtained surface height histograms (Figure 4c). The obtained result shows the significant potential of Ag-mesh combined with PEDOT:PSS as a TCE for large-area optoelectronic devices and underlines the proper choice of polymer ink formulations to ensure full control over uniform coatings.

## 3 Conclusions

In this contribution, we demonstrate a novel approach for manufacturing transparent conductive electrodes (TCEs) onto a flexible substrate using the XTPL ultraprecise deposition technique, which significantly reduces production costs and simplifies the whole deposition process in contrast to well-known lithography and vacuum deposition processes. The obtained Ag metal-mesh electrode onto a flexible substrate NOA63 exhibits an outstanding sheet resistance of 11 Ω/sq, maintaining the optical transmittance at 80% (λ = 550 nm). The QLED obtained on top of the presented Ag-mesh electrode resulted in uniform emission with a maximum at 515 nm, a maximum external quantum efficiency (EQE) of 2%, and a current efficiency (CE) of 6 cd/A, reaching the maximum luminance (L) of 3200 cd/m^2^ at a current density of 100 mA/cm^2^. The fact that the whole process does not require any special preparation or demanding equipment (e.g., vacuum deposition, lithography, etc.) makes it an interesting example of rapid and easy device prototyping.

## 4 Methods

### 4.1 Silver Metal-Mesh TCE Fabrication and Characterization

First, the silver metal mesh is printed by the XTPL UPD method onto the glass substrate. The used ink was composed of 82 wt % silver content. The printed mesh is then sintered into a hot plate at 250 °C for 30 min in air. Next, a drop of liquid photopolymer NOA63 is deposited in each corner and in the middle of the printed Ag-mesh. NOA63 is a commercial ultraviolet (UV) curable polymer of Norland optical adhesive, which serves as a transparent substrate. The PET foil is pressed against the substrate in order to uniformly spread the deposited NOA63.

The photopolymer film is cured by 365 nm UV irradiation at room temperature for 10 min. As a result, the silver mesh is embedded in the cross-linked NOA63. Then, the PET foil is peeled off from the substrate. Subsequently, NOA63 with the silver mesh is peeled off from the glass substrate and cut into the desired size.

The silver metal mesh was imaged by scanning electron microscopy (SEM/Ga-FIB Microscope FEI Helios NanoLab 600i). The optical transmittance was measured by UV–vis spectroscopy within the wavelength range of 200–1100 nm. For the electrical resistance measurement, the Ag metal mesh was prepared with laterally displaced contact pads on each side of the printed mesh and simultaneously sintered (hot plate 250 °C for 30 min in the air). The pads were made with the same ink as the silver mesh. The electrical resistance between the contact pads is equivalent to the sheet resistance when the conductor layer is square, as in the case of the particular experiment. The electrical resistance for a single Ag line was determined in the same way as that for the Ag metal mesh. The morphology of the obtained Ag metal mesh, as well as of the single Ag line, was measured by a confocal microscope (Olympus OLS5100).

### 4.2 QLED Fabrication and Characterization

Green-emitting QDs (CdSe@ZnS/ZnS) emitting at 515 nm (PLQY ∼ 55%; fwhm, 21 nm) were synthesized according to a previously described method.^19^ The QLED structure was deposited on top of a Ag metal-mesh TCE in a nitrogen-filled glove box. First, the isopropanol solution of PETOD:PSS (HTL Solar 3, Ossila Ltd.) was first spin-coated at 4000 rpm for 60 s and annealed at 90 °C for 20 min. Then, TFB solution (10 mg/mL in chlorobenzene) was deposited at 3000 rpm for 30 s and annealed at 110 °C for 20 min. The QD solution (10 mg/mL in octane) was spin-coated at 3000 rpm without any annealing. The electron-transport layer was deposited by spin-coating ZnMgO (5% mol Mg) NP solution (20 mg/mL in ethanol) and annealed at 90 °C for 10 min. Finally, the Al electrode (99.99%) was sputtered in Ar plasma at a pressure of 10^–2^ mbar to yield a pixel area of 2.25 mm^2^. The device characteristics were measured using the setup composed of a Konica Minolta LS-160 luminance meter coupled with a Keithley 2400 source-meter and FLAME–S-UV–vis spectrometer to collect the electroluminescence spectrum. The morphology of the TCE was determined using a Park System XE-100 atomic force microscope (AFM) working in tapping mode.

## References

[ref1] Yao L.; Qin Y.; Li X.; et al. High-efficiency stretchable organic light-emitting diodes based on ultra-flexible printed embedded metal composite electrodes. InfoMat 2023, 5 (5), e12410 10.1002/inf2.12410.

[ref2] Li D.; Lai W.; Zhang Y.; et al. Printable transparent conductive films for flexible electronics. Adv. Mater. 2018, 30 (10), 1704738 10.1002/adma.201704738. 29319214

[ref3] Murawski C.; Gather M. C. Emerging biomedical applications of organic light-emitting diodes. Adv. Opt. Mater. 2021, 9 (14), 2100269 10.1002/adom.202100269.

[ref4] Morales-Masis M.; De Wolf S.; Woods-Robinson R.; et al. Transparent electrodes for efficient optoelectronics. Adv. Electron. Mater. 2017, 3 (5), 1600529 10.1002/aelm.201600529.

[ref5] Gedda M.; Das D.; Iyer P. K.; et al. Work function tunable metal-mesh based transparent electrodes for fabricating indium-free organic light-emitting diodes. Mater. Res. Express 2020, 7 (5), 054005 10.1088/2053-1591/ab8d5b.

[ref6] Fischer R.; Gregori A.; Sahakalkan S.; et al. Stable and highly conductive carbon nanotube enhanced pedot:pss as transparent electrode for flexible electronics. Org. Electron. 2018, 62, 351–356. 10.1016/j.orgel.2018.07.022.

[ref7] Yu L.; Shearer C.; Shapter J. Recent development of carbon nanotube transparent conductive films. Chem. Rev. 2016, 116 (22), 13413–13453. 10.1021/acs.chemrev.6b00179. 27704787

[ref8] Sharma S.; Shriwastava S.; Kumar S.; et al. Alternative transparent conducting electrode materials for flexible optoelectronic devices. Opto-Electron. Rev. 2018, 26 (3), 223–235. 10.1016/j.opelre.2018.06.004.

[ref9] Govind R. K.; Mondal I.; Baishya K.; et al. Large-area fabrication of high performing, flexible, transparent conducting electrodes using screen printing and spray coating techniques. Adv. Mater. Technol. 2022, 7 (6), 2101120 10.1002/admt.202101120.

[ref10] Jeon I.; Yoon J.; Ahn N.; et al. Carbon nanotubes versus graphene as flexible transparent electrodes in inverted perovskite solar cells. J. Phys. Chem. Lett. 2017, 8 (21), 5395–5401. 10.1021/acs.jpclett.7b02229. 28994283

[ref11] Aleksandrova M.; Kurtev N.; Videkov V.; et al. Material alternative to ito for transparent conductive electrode in flexible display and photovoltaic devices. Microelectron. Eng. 2015, 145, 112–116. 10.1016/j.mee.2015.03.053.

[ref12] Lian L.; Xi X.; Dong D.; et al. Highly conductive silver nanowire transparent electrode by selective welding for organic light emitting diode. Org. Electron. 2018, 60, 9–15. 10.1016/j.orgel.2018.05.028.

[ref13] Ghosh D. S.; Chen T. L.; Pruneri V. High figure-of-merit ultrathin metal transparent electrodes incorporating a conductive grid. Appl. Phys. Lett. 2010, 96 (4), 041109 10.1063/1.3299259.

[ref14] Jang Y.; Kim J.; Byun D. Invisible metal-grid transparent electrode prepared by electrohydrodynamic (EHD) jet printing. J. Phys. D: Appl. Phys. 2013, 46 (15), 155103 10.1088/0022-3727/46/15/155103.

[ref15] Jung E.; Kim C.; Kim M.; et al. Roll-to-roll preparation of silver-nanowire transparent electrode and its application to large-area organic light-emitting diodes. Org. Electron. 2017, 41, 190–197. 10.1016/j.orgel.2016.11.003.

[ref16] Nam S.; Song M.; Kim D. H.; et al. Ultrasmooth, extremely deformable and shape recoverable ag nanowire embedded transparent electrode. Sci. Rep. 2014, 4 (1), 4788 10.1038/srep04788. 24763248 PMC3999473

[ref17] Chrzanowski M.; Kliczkowski M.; Bieganski P.; et al. Enhanced transparency of Ultrathin AG films through wetting layer of phosphomolybdic acid. Thin Solid Films 2020, 694, 137734 10.1016/j.tsf.2019.137734.

[ref18] Chrzanowski M.; Banski M.; Podhorodecki A. Semi-transparent quantum-dot light-emitting diode with ultrathin au electrode embedded in solution-processed coatings. Mater. Lett. 2019, 257, 126709 10.1016/j.matlet.2019.126709. PMC9062623 35515297

[ref19] Chrzanowski M.; Banski M.; Sitarek P.; et al. Quantum-dot light-emitting diode with ultrathin au electrode embedded in solution-processed phosphomolybdic acid. RSC Adv. 2019, 9 (19), 10754–10759. 10.1039/C9RA01680E. 35515297 PMC9062623

[ref20] Zhou L.; Xiang H. Y.; Shen S.; et al. High-performance flexible organic light-emitting diodes using embedded silver network transparent electrodes. ACS Nano 2014, 8 (12), 12796–12805. 10.1021/nn506034g. 25470615

[ref21] Chen X.; Guo W.; Xie L.; et al. Embedded AG/Ni metal-mesh with low surface roughness as transparent conductive electrode for optoelectronic applications. ACS Appl. Mater. Interfaces 2017, 9 (42), 37048–37054. 10.1021/acsami.7b11779. 28967742

[ref22] Han J. W.; Jung B.; Kim D. W.; et al. Transparent conductive hybrid thin-films based on copper-mesh/conductive polymer for ITO-free organic light-emitting diodes. Org. Electron. 2019, 73, 13–17. 10.1016/j.orgel.2019.05.018.

[ref23] Łysień M.; Witczak Ł.; Wiatrowska A.; et al. High-resolution deposition of conductive and insulating materials at micrometer scale on complex substrates. Sci. Rep. 2022, 12 (1), 9327 10.1038/s41598-022-13352-5. 35665755 PMC9167286

[ref24] Wiatrowska A.; Kowalczewski P.; Fiączyk K.; et al. 59–3: Ultra-precise printing of micrometer-size interconnectors for high-resolution microled displays. SID Symp. Dig. Tech. Pap. 2021, 52 (1), 833–836. 10.1002/sdtp.14812.

[ref25] Wiatrowska A.; Kowalczewski P.; Fiączyk K.; Witczak Ł.; Gadzalińska J.; Łysień M.; Schneider L.; Granek F. 27.3: Ultraprecise deposition of micrometer-size conductive structures for printed displays. SID Symp. Dig. Tech. Pap. 2021, 52 (S2), 370 10.1002/sdtp.15126.

